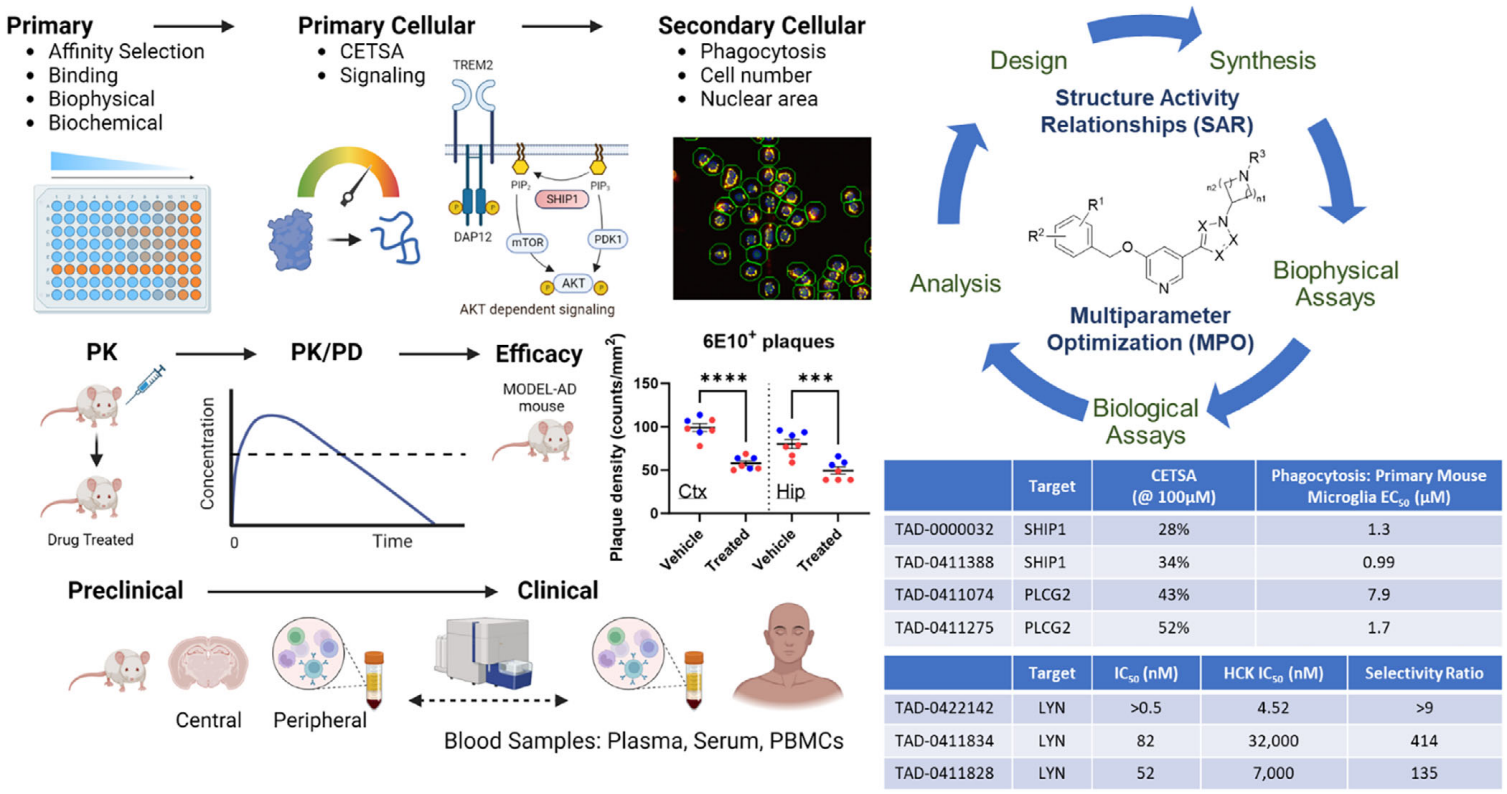# IUSM‐Purdue TREAT‐AD Center Capabilities and Strategy to Enable and Advance Novel Therapeutic Targets for the Treatment of Alzheimer’s Disease

**DOI:** 10.1002/alz.087660

**Published:** 2025-01-09

**Authors:** Stephanie A. Mowery, Kun Huang, Andrew D. Mesecar, Jeffrey L. Dage, Brent Clayton, Bruce T. Lamb, Alan D. Palkowitz, Timothy I. Richardson

**Affiliations:** ^1^ Indiana Biosciences Research Institute, Indianapolis, IN USA; ^2^ Indiana University, School of Medicine, Indianapolis, IN USA; ^3^ Purdue, West Lafayette, IN USA; ^4^ Indiana University School of Medicine, Indianapolis, IN USA

## Abstract

**Background:**

The TaRget Enablement to Accelerate Therapy Development of Alzheimer’s Disease (TREAT‐AD) Centers are dedicated to identifying and validating targets from the NIH Accelerating Medicines Partnership for Alzheimer’s Disease (AMP‐AD). The centers develop Target Enabling Packages (TEPs) to explore new therapeutic target hypotheses, moving beyond the traditional focus on amyloid or tau pathologies. In accordance with open science principles, data, methods, and tools are freely shared with the research community via an open‐access platform, the AD Knowledge Portal. The Indiana University School of Medicine and Purdue University TREAT‐AD (IUSM Purdue TREAT‐AD) Center comprises four technical cores: Bioinformatics and Computational Biology (BCB), Structural Biology and Biophysics Core (SBB), Assay Development and High Throughput Screening (ADHTS), and Medicinal Chemistry and Chemical Biology (MCCB). These cores collaborate to develop research tools that are used to validate biological targets and assess their druggability with an initial focus on understanding the role of neuroinflammation in AD.

**Method:**

The BCB Core supports target selection and validation with data and analysis. The SBB Core provides proteins for assay development, biophysical assays, and structural studies to aid in mode of action and Structure Activity Relationship (SAR) studies. The ADHTS Core develops in vitro and in vivo assays for SAR studies and translational PD biomarker strategies to assist in determining early phase clinical dosing regimens. The MCCB Core selects therapeutic modalities (small molecules, antibodies, siRNA) and discovers pharmacological tools, employing strategies for SAR studies to balance pharmacological and drug‐like properties.

**Result:**

Target Enabling Packages (TEPs) are now available via the AD Knowledge Portal for microglia targets that were prioritized for early drug discovery studies. TEPs include bioinformatics analysis, biological reagents and protocols, protein production methods, and recommended chemical probes with detailed information (Figure 1). Novel small molecule hits and leads were identified for SHIP1, PLCG2, SHP1 and LYN/HCK.

**Conclusion:**

A pipeline of prioritized microglia targets were selected and enabled for early drug discovery. The IUSM Purdue TREAT‐AD Center is now working with AMP‐AD researchers to explore biological hypothesis in addition to the role of neuroinflammation in AD.